# Decoding Aging through iPSC Reprogramming: Advances and Challenges

**DOI:** 10.14336/AD.2025.0438

**Published:** 2026-05-05

**Authors:** Rui-lin Li, Yun-zeng Zou, Sheng Kang

**Affiliations:** ^1^Department of Cardiovascular Medicine, Shanghai East Hospital, School of Medicine, Tongji University, Shanghai 200120, China.; ^2^Department of Cardiology, Shanghai Institute of Cardiovascular Diseases, Zhongshan Hospital, Fudan University, Shanghai 200032, China.

**Keywords:** Induced pluripotent stem cells, Cellular reprogramming, Aging, Epigenetic memory

## Abstract

Aging is characterized by cellular senescence and increased susceptibility to age-related diseases. Induced pluripotent stem cell (iPSC) technology demonstrates the potential to reverse aging hallmarks, including telomere attrition, mitochondrial dysfunction, and oxidative stress. Reprogramming somatic cells using factors such as Oct4, Sox2, Klf4, and c-Myc (OSKM) restores pluripotency and reverses aging markers. Partial reprogramming, involving transient OSKM expression, rejuvenates cells by resetting epigenetic clocks, reducing senescence-associated secretory phenotypes (SASPs), and improving mitochondrial function, as evidenced by lifespan extension in progeroid mouse models. These advancements facilitate disease modeling and autologous therapies for neurodegeneration, etc. Critical challenges, including tumorigenicity risks associated with oncogenic reprogramming factors, have been mitigated through non-integrative delivery systems (e.g., mRNA, small molecules) and suicide genes. Persistent epigenetic memory and incomplete reprogramming impede iPSC differentiation, but CRISPR-based tools (e.g., dCas9-DNMT3A, CRISPRoff) allow precise epigenetic editing to erase residual somatic signatures. Variability in iPSC quality, influenced by cell source and culture conditions, necessitates standardized protocols and CRISPR-enhanced quality control. Ethical considerations, such as informed consent and genetic discrimination, highlight the need for governance frameworks that align innovation with societal values. Subsequent priorities include optimizing reprogramming efficiency, validating safety in preclinical models, and translating findings into therapies for age-related disorders. In conclusion, iPSC and CRISPR technologies collectively present transformative strategies to delay aging and restore cellular vitality, paving the way for rejuvenation therapies. Future studies should focus on improving the reprogramming efficiency, minimizing the risk of tumorigenicity, and exploring the optimized CRISPR-based epigenetic editing technique.

## Introduction

**Figure 1.**
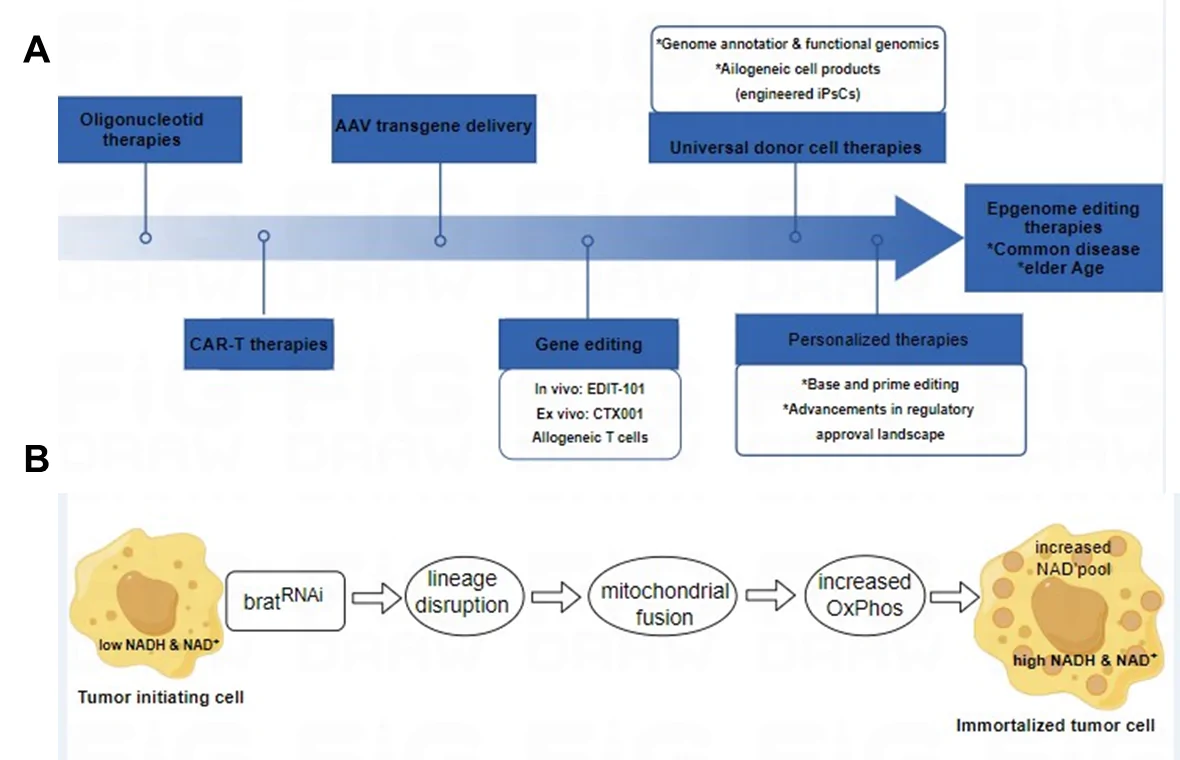
**Outlining the development of gene therapy for aging and immortalization. (A)** Timeline shows the significant events leading up to gene therapy for common diseases and aging. **(B)** The study definitively shows that mitochondrial fusion promotes neural stem cell immortalization via oxidative phosphorylation and NAD+ metabolism. It is achieved by using single-cell transcriptomics and cellular lineage tracing, which are induced by knocking down the transcription factor Brat. The study also uses the targeted metabolomics and in vivo genetic screening. Immortalization refers to the acquisition of indefinite proliferative capacity, bypassing replicative senescence.

Aging involves a variety of biological processes marked by the deterioration of physiological integrity and an increased risk of illness and death. Cellular senescence, characterized by a progressive loss of vitality and an increased vulnerability to pathological states, is a critical risk in aging [1]. The advent of iPSCs technology has provided novel avenues for addressing the cellular impacts of aging (Fig. 1A).

## Main Findings

### iPSC Technology

iPSCs are somatic cells that have been reprogrammed to revert to an embryonic stem cell-like state, thereby regaining pluripotency and the capacity for differentiation into any cell types within the body [2]. The breakthrough technology holds significant promise for regenerative medicine and the treatment of age-related ailments. The introduction of specific transcription factors, such as Oct4, Sox2, Klf4, and c-Myc (OSKM), is critical for reprogramming somatic cells into iPSCs. These factors can reset the cellular identity and reverse the accumulation of aging markers [3].

One of the most notable aging hallmarks that iPSC technology has been shown to reverse telomere shortening. Telomeres are the protective caps at the ends of chromosomes that shorten with each cell division, ultimately inducing cellular senescence. iPSCs exhibit the elongated telomeres compared to their somatic cell precursors, suggesting a potential for prolonged cellular lifespan [4]. Furthermore, iPSC technology also addresses mitochondrial dysfunction. Mitochondria are the engines of cells, and their decline in function is a well-documented aspect of aging. Studies have shown that iPSCs have improved mitochondrial function, including the increased adenosine triphosphate (ATP) production and the reduced oxidative stress, which are both indicative of a more youthful cellular state [5]. Moreover, the metabolic reprogramming, including mitochondrial fusion, the increased oxidative phosphorylation and nicotinamide adenine dinucleotide (NAD⁺) production, and the excessive NAD^+^ formation triggered tumor cell immortalization (Fig. 1B) [6].

The potential of iPSC technology to treat age-related disorders is investigated, for example, iPSCs derived from patients with neurodegenerative diseases are used to model the disease in vitro, thereby providing valuable insights into disease mechanisms and potential therapeutic targets [7]. Additionally, the application of iPSCs in autologous cell therapy presents an exciting opportunity for personalized medicine, wherein the patient's own cells can be reprogrammed, differentiated into the required cell type, and subsequently reintroduced into their body to replace damaged or senescent cells [8].

### Partial Reprogramming and Reversal of Age-Associated Phenotypes

**Figure 2.**
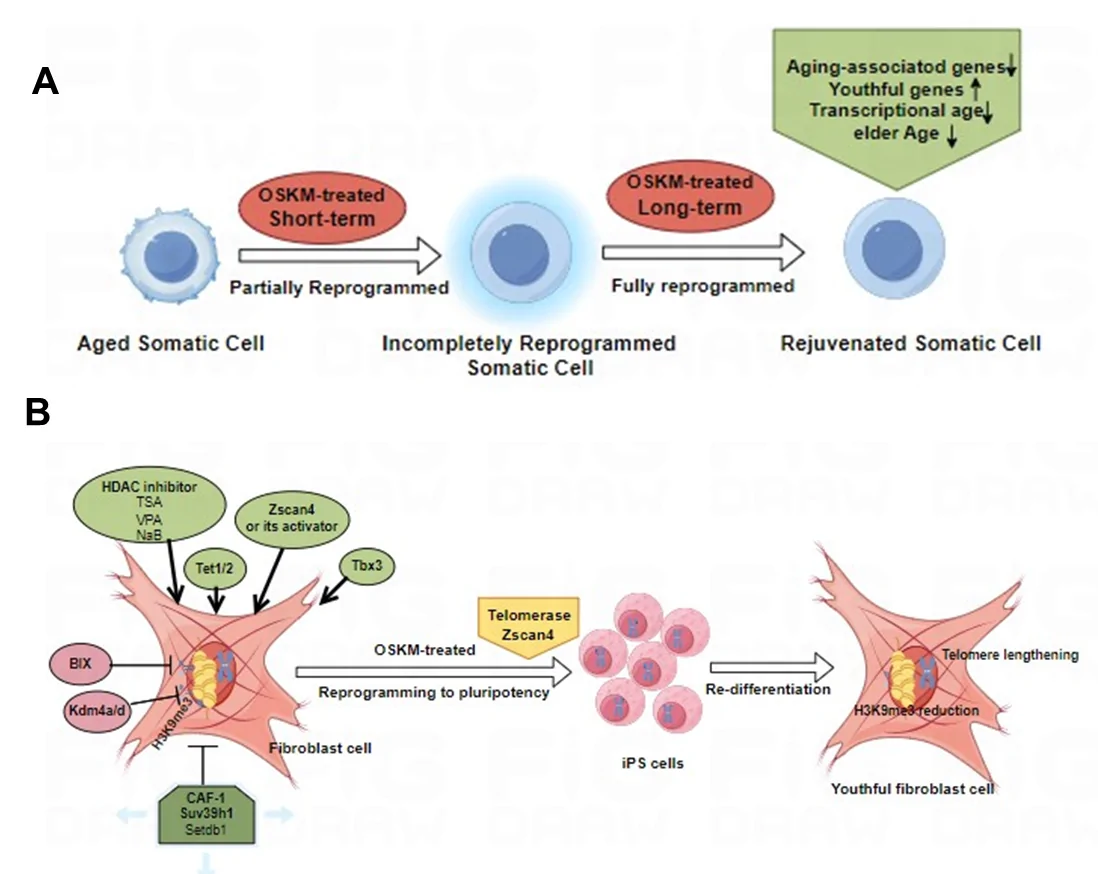
**Youthful Gene and Telomere are changed in Cellular Reprogramming Process. (A)** The graph illustrates the changes of cellular markers before and after partially reprogramming and fully reprogramming, showing the improvements of youthful gene expression. OSKM denotes Oct4, Sox2, Klf4, and cMyc. Youthful Gene refers to genes involving in telomere maintenance, mitochondrial efficiency, epigenetic regulation, and oxidative stress reduction, whose expression or activity is associated with younger cellular phenotypes, the reduced senescence markers and the improved cellular function. **(B)** Telomere lengthening and maintenance in iPSCs required both telomerase and sporadic Zscan4 expression. Telomere elongation and somatic cell reprogramming in iPSC were enhanced by reprogramming facilitators involving DNA demethylation (AZA, Tet1/2, and Tbx3), histone acetylation (TSA, VPA, and NaB), Zscan4 or its activator, and H3K9me3/2 demethylation (BIX, Kdm4a/d). Suv39h1, Setdb1, and CAF-1 were H3K9me3 methyltransferases that inhibited telomere extension and reprogramming. CAF-1, chromatin assembly factor-1; iPSCs, induced pluripotent stem cells; Tbx3, T-box 3; Tet1/2, Ten-eleven Translocation 1/2; VPA, valproic acid.

The partial reprogramming process is designed to transiently stimulate the expression of key reprogramming factors, such as the Yamanaka factors, for a limited duration. The transient stimulation leads to the amelioration of cellular aging hallmarks, including the restoration of telomere length, improving mitochondrial activity (e.g., increased ATP production), resetting epigenetic clocks (e.g., DNA methylation patterns), reducing senescence-associated secretory phenotypes (SASPs) and the reversion of cellular epigenetic states towards a more youthful profile [3, 9,10]. The transient nature of the reprogramming factors is essential for both telomere elongation and the induction of a fully pluripotent state, which improves youthful genes expression and telomere lengthening (Fig. 2A and Fig. 2B) [11].

One of the most convincing examples of the possibilities of partial reprogramming is its impact on progeroid mouse models. For example, those by Ocampo et al. showed that partial reprogramming led to the rejuvenation of cells (improving cellular function, reducing senescence, and reversing age-related molecular changes) and an extension of the lifespan in the models [3].Furthermore, the role of specific epigenetic regulators and the interactions between the reprogramming factors and the cellular micro-environment are fields of ongoing investigation [12].

By reversing age-associated phenotypes, the partial reprogramming can potentially slow down the progression of diseases such as Alzheimer's, Parkinson's, and cardiovascular diseases [13]. As the field of cellular programming and aging continues to evolve, future studies will focus on several important topics as it develops, i.e., elucidating the precise molecular pathways involved in the amelioration of age-associated phenotypes through partial reprogramming, understanding the long-term effects of the approach on cellular function and organ health, and translating these findings into safe and effective therapeutic strategies for human use.

## Challenges

### Tumorigenicity Risks in Long-Term Safety

Tumorigenicity risk arises from the potential of reprogrammed cells to form tumors as a result of their enhanced proliferative performance. The reprogramming factors, such as OSKM, which are essential for inducing pluripotency, can also trigger the acquisition of a neoplastic phenotype if not properly controlled [14,15]. Additionally, the incomplete epigenetic reprogramming may cause cells to retain memory of their initial differentiated state, which eventually affects their functionality and safety in the long term [16]. Furthermore, there is a growing emphasis on understanding the long-term behavior of reprogrammed cells in vivo, including their differentiation potential and stability over time [17].

Recent research has focused on strategies to reduce the tumorigenicity risk associated with cellular reprogramming. One approach involves the use of elastin-like polypeptide-based methods to deliver reprogramming factors, which reduces the risk of genomic instability and insertional mutagenesis [18,19]. Additionally, the development of small molecules and chemical cocktails that induce pluripotency without the use for viral vectors is becoming increasingly popular as a safer alternative [20].

Another critical strategy to handle tumorigenicity is the implementation of strict monitoring and control measures. These include the use of in vitro assays to assess the proliferative ability of reprogrammed cells and the expression of tumor suppressor genes [21]. Furthermore, the development of safety measures, such as the incorporation of suicide genes that can be triggered to destroy reprogrammed cells in the case of uncontrolled growth, is also investigated [22].

**Figure 3.**
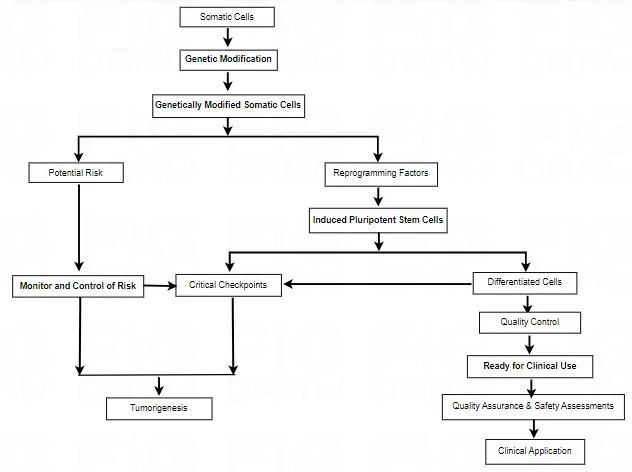
**A Schematic of Key Checkpoints is presented in Cellular Reprogramming Process**. It detailed the stages from genetic modification of somatic cells to iPSC differentiation, with emphasis on critical checkpoints and potential risks of tumorigenesis.

Animal model-based preclinical studies are strikingly important in evaluating the long-term safety of reprogramming technologies, which provide valuable insights into the behavior of iPSCs and their derivatives in a living organisms and help to predict potential risks in patients [23]. As iPSC-based therapies move towards clinical trials, it is crucial to carefully monitor and assess their safety and efficacy in patients, with a focus on potential long-term effects [24]. To supervise the therapeutic use of reprogrammed cells, it is necessary to establish robust regulatory frameworks and conduct a meticulous case-by-case analysis of the risks and benefits (Fig. 3) [25,26].

### Reprogramming Efficiency

Recent advancements have been made to improve the efficiency of the reprogramming process, which include the optimization of reprogramming factors, the use of small molecules to replace or supplement the traditional viral vectors, and the creation of non-integrative delivery techniques, i.e., mRNA [27,28].

Despite improvements in efficiency, the variability in the quality of iPSCs remains a remarkable challenge. The variability is attributed to differences in the initial somatic cell type, the reprogramming agents use, and the culture conditions [29]. The technical challenges, such as iPSC lineage fidelity, reprogramming-induced mutations, or model limitations in reflecting human aging, were presented in Table 1.

**Table 1.** Technical Challenges and Barriers to Clinical Translation in iPSC Reprogramming.

Technical Challenge	Explanation	Examples	Clinical Implications
**Lineage Fidelity**	Residual epigenetic memory biases differentiation toward the original somatic lineage.	Fibroblast-derived iPSCs retain stromal signatures, skewing neuronal differentiation.	Misdifferentiated cells may cause functional failures (e.g., arrhythmias, tumor formation).
**Reprogramming-Induced Mutations**	Genomic instability during reprogramming introduces oncogenic mutations.	Copy-number variations (CNVs) in c-MYC and point mutations in TP53 due to oxidative stress.	Tumorigenic risk in transplanted cells compromised safety of iPSC-derived therapies.
**Heterogeneity in iPSC Lines**	Variability across iPSC lines arises due to donor age, genetic background, and culture conditions.	Batch-to-batch differences in differentiation efficiency and epigenetic profiles.	Challenges in reproducibility, scalability, and regulatory approval for therapies.
**Murine Model Limitations**	Mice inadequately replicate human aging owing to biological and physiological differences.	Mice retain telomerase activity in somatic cells. Short lifespan limits chronic aging studies.	Poor translation of murine results in human aging and unreliable drug testing outcomes.
**Functional Immaturity**	iPSC-derived cells often exhibit fetal-like, immature phenotypes.	Cardiomyocytes lack T-tubules; neurons show the limited synaptic connectivity.	Potential failure of integration and function in immature cells reduces therapeutic efficacy.
**Immune Compatibility**	Autologous iPSCs may trigger immune responses due to residual immunogenicity.	Minor HLA mismatches or epigenetic aberrations in iPSCs activate T-cell responses.	Risk of rejection requires immunosuppression or universal "off-the-shelf" iPSC lines

In future, the microphysiological systems (MPS), also known as "organ-on-a-chip" platforms, show significant potential to better replicate human aging physiology compared to traditional models like mice. Standardizing protocols, advancing CRISPR-based quality control, and prioritizing human-centric models will accelerate the transition from bench to bedside [30]. As the field evolves, interdisciplinary collaboration integrating genomics, bioengineering, and artificial intelligence will be key to overcoming these barriers.

## Epigenetic Barriers: Incompleteness and Memory

**Figure 4.**
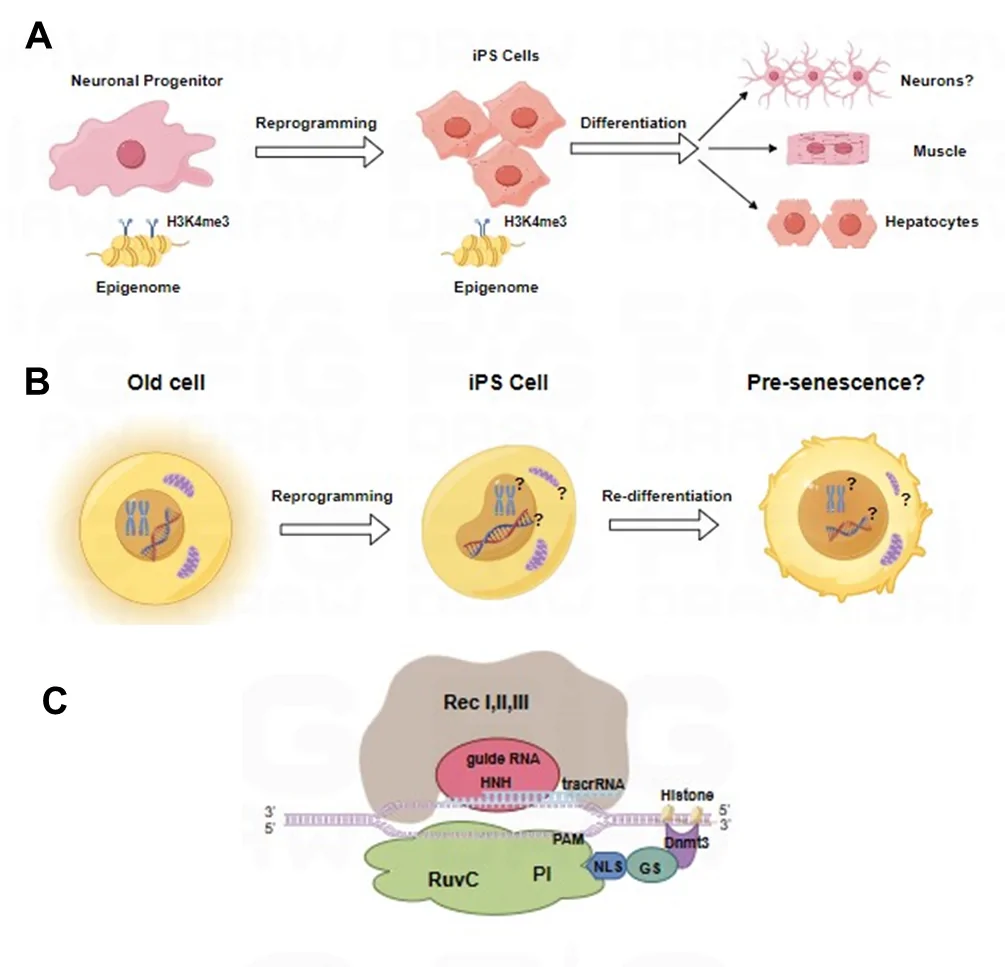
**Epigenetic Memory and Aging in Cellular Reprogramming Process. (A)** Reprogramming and epigenetic memory: iPSCs undeniably retain certain epigenetic marks from somatic precursors. Residual epigenetic memory can partially reactivate pluripotency genes, maintain lineage-specific markers, and retain age-related epigenetic signatures. **(B)** Reprogramming and aging-related aspects: Reprogramming does not fully rejuvenate aged somatic cells or cure age-related changes such as DNA damage, telomere shortening, and mitochondrial dysfunction. iPSCs derived from old somatic cells are prone to early senescence. **(C)** The graph illustrates CRISPR/dCas9 targeting age-related epigenetic marks (e.g. DNA methylation). sgRNA guides dCas9 to the target genomic region (usually a CpG island or promoter region) and identifies the target DNA sequence (PAM sites, such as NGG of SpCas9). The fused methyltransferase (such as the DNMT3A catalytic domain) catalyses the methylation of cytosine C near the binding site to form 5-methylcytosine (5 mC), which usually occurs on the cytosine of the CpG dinucleotide. CpG methylation in promoter regions suppresses gene transcription. It is achieved by hindering transcription factor binding or recruiting histone modifiers. The result is epigenetic silencing of target genes. Dnmt3, DNA Methyltransferase 3; GS, Gene Silencing; HNH, Holliday Junction Nuclease Homology; NLS, Nuclear Localization Signal; PAM, Protospacer Adjacent Motif; PI, Phosphatase/Protease Inhibitor; Rec I/II/III, Recombination Domain I/II/III; Ruv C, Resolution of Holliday Junctions; sgRNA, single guide RNA; tracrRNA, trans-activating CRISPR RNA.

Despite the noticeable ability of reprogramming factors to induce a pluripotent state in somatic cells, the process does not fully erase all epigenetic marks accumulated during the cell's lifetime. This phenomenon, termed "epigenetic incompleteness," results in the incomplete rejuvenation of cells [31]. The residual epigenetic memory leads to partial reactivation of pluripotency genes, persistence of lineage-specific markers, and even the retention of certain age-related epigenetic signatures (Fig. 4A and Fig. 4B) [32]. Importantly, the incomplete epigenetic reprogramming keeps iPSCs with memory of their somatic origin, which influences their differentiation potential and contributes to variability [16]. Recent studies have focused on improving the reprogramming protocols to achieve more complete epigenetic resetting and produce higher-quality iPSCs [33]. Additionally, the combination of computational models and machine learning algorithms (artificial intelligence) may provide insights into the intricate interactions of factors that influence reprogramming efficiency and iPSC quality [31].

Recent research has focused on optimizing the reprogramming process to more effectively reset the epigenetic landscape of somatic cells. One approach involves the use of additional epigenetic modifiers or tiny chemicals that target specific histone modifications, aiming to enhance the removal of somatic epigenetic marks [32]. Another strategy is to lengthen the reprogramming period or to use a progressive reprogramming approach, which allows for a more thorough reconfiguration of the epigenome [34].

Incredibly, the incompleteness of epigenetic reprogramming has significant implications for the studies of aging and the advancement of therapies for age-related ailments. The epigenetic memory in iPSCs derived from aged or sick cells provides valuable insights into the epigenetic basis of ageing and disease pathogenesis [35]. Moreover, a better understanding of the factors that contribute to epigenetic incompleteness will lead to novel therapeutic strategies that target the epigenetic regulation of gene expression in ageing and illness. In future, novel cutting-edge technologies, such as CRISPR-based epigenetic editing tools, may bring innovative solutions for precise and comprehensive epigenetic reprogramming [36].

Noticeably, epigenetic memory refers to the phenomenon where iPSCs retain certain epigenetic marks from their somatic cell precursors, despite the reprogramming process, which preserves signatures including DNA methylation patterns and histone modifications that are unique to the original cell type. The presence of epigenetic memory potentially affects the differentiation potential of iPSCs, as well as their behavior in vivo, which may have implications for their therapeutic use [37].

The debate surrounding epigenetic memory focused on whether it was a beneficial or detrimental aspect of iPSC biology (Fig. 4A and Fig. 4B) [38]. Some researchers argued that epigenetic memory could be used to guide the differentiation of iPSCs into specific cell lineages, potentially improving the efficiency of cell-based therapies [39]. However, others considered that the presence of epigenetic memory might result in aberrant functions in reprogrammed cells. This would raise the risk of unpredictable behaviors, such as tumorigenicity, when these cells were employed in regenerative medicine applications [37]. Inevitably, the contradictions in the literature on iPSCs mainly stem from the specific variables and methodological differences. Both articles clearly highlight the epigenetic memory in iPSCs, where reprogrammed cells retain methylation signatures of their origin tissue, but this varies among studies [32,38]. For example, iPSCs may retain epigenetic memory of their tissue of origin, which could influence their differentiation potential [32]. Conversely, iPSCs can maintain tissue-specific methylation memory even at high passage numbers [38]. iPSCs derived from different somatic cells may exhibit varying degrees of functional maturity, i.e., blood-derived iPSCs show more blood-forming potential than fibroblast-derived iPSCs [32]. On the other hand, iPSC-derived cells exhibit functional defects such as premature senescence [38]. Thus, the variations in experimental protocols and evaluation methods across different studies may contribute to the observed discrepancies in results. Future studies should standardize protocols, develop tissue - specific strategies, and set benchmarks for maturity and safety.

Recent advances have shed light on the mechanisms underlying epigenetic memory and its impact on iPSC functioning. High-throughput sequencing methods, such as whole-genome bisulfite sequencing and chromatin immunoprecipitation sequencing, have enabled a more comprehensive analysis of the epigenetic landscape of iPSCs [40,41]. According to these studies, some epigenetic marks, especially those in heterochromatic regions, may remain during reprogramming, even while many other marks are reset. Certainly, Table 2 clearly shows the relationship between partial reprogramming, epigenetic memory, and cellular senescence.

**Table 2.** The relationship between Partial Reprogramming, Epigenetic Memory, and Cellular Senescence.

Relationship Aspect	Model Organisms/Cell Types	Reprogramming Duration	Key Findings	Measurable Outcomes	References
**Partial Reprogramming and Cellular Senescence**	Progeroid mice (Lmna G609G mutant), human fibroblasts	Cyclic induction (2 days ON, 5 days OFF for 6 weeks)	Transient OSKM exposure reduces senescence markers, restores mitochondrial function, and extends lifespan.	30% lifespan extension; decrease in SA-β-gal activity; increase in muscle regeneration capacity	[3, 59]
**Epigenetic Memory and Cellular Senescence**	Yeast Strains, iPSCs (e.g., fibroblasts, neural progenitors, hematopoietic cells), etc	Reprogramming duration depends on donor age and cell type, etc.	Lineage-specific epigenetic memory biases differentiation efficiency and retains age-associated marks.	Some studies found residual methylation signatures in iPSCs, while others observed attenuation with passage number.	[38, 32]
**Partial Reprogramming and Epigenetic Memory**	Aged human fibroblasts	Total reprogramming time is approximately 20 days in mice and 30 days in humans	Partial reprogramming resets DNA methylation clocks while retaining somatic cell identity.	Reduction in epigenetic age (Horvath clock); Maintenance of fibroblast markers (e.g., Vimentin)	[60, 61]
**Cellular Senescence and Epigenetic Regulation**	Progeroid mice (Lmna G609G mutant), human fibroblasts, murine fibroblasts, iPSCs, etc	No specific descriptions of reprogramming duration	SUV39H1 loss exacerbates SASP; H3K9me3 restoration delays senescence.	Increase IL-6, IL-8 (SASP factors); reduce heterochromatin (H3K9me3); increase SA-β-gal activity in senescent cells	[62, 63]

To tackle the possible problems of epigenetic memory, researchers explored strategies to enhance the completeness of epigenetic reprogramming, which involved the use of small molecules that target epigenetic modifiers, such as DNA methyltransferase inhibitors and histone deacetylase inhibitors. These molecules facilitate a more thorough removal of somatic cell epigenetic marks [42]. Additionally, ongoing studies were also conducted on the use of non-integrative reprogramming methods, such as the use of episomal vectors or transient mRNA delivery. This is to minimize the risk of insertional mutagenesis and improve the epigenetic resetting of reprogrammed cells [28].

## CRISPR-Based Epigenetic Editing

Recent advances in clustered regularly interspaced short palindromic repeats (CRISPR) technology have introduced novel tools for targeted epigenetic modulation. These offer a promising solution to the challenges of incomplete reprogramming and residual epigenetic memory. It enables precise epigenetic editing (e.g., DNA methylation, histone acetylation) to address incomplete reprogramming and residual epigenetic memory (Fig. 4C). CRISPR-based epigenetic editing is the most precise method for controlling the expression of genes related to cellular reprogramming [43]. Also, it uses single guide RNAs to direct a fusion protein of nuclease-deficient Cas9 with DNMT3A/3L to specific sites in the genome, enabling site-specific modification of DNA methylation patterns. DNMT3A-mediated epigenome editing is transient in most regions and is often lost within a few days. More complex fusion proteins, such as CRISPRoff, have been developed to increase the stability of DNA hypermethylation and achieve more sustained epigenetic clock resetting effects [44]. Similarly, the specificity of CRISPR-based transcription activators modulated the epigenetic marks of the TERT promoter, and induced telomerase expression. Therefore, the strategy of cell immortalization could be potentially adopted and generalized to delay cell death or even immortalize any other cell types [45]. Importantly, dCas9 can be coupled with histone acetyltransferases like p300 to increase histone acetylation at the promoter regions of genes such as IL1RN, MYOD, and OCT4. This will lead to significant transcriptional activation of the corresponding genes. Conversely, dCas9 fused with histone deacetylases decreases acetylation at promoter regions, thereby repressing gene expression. It provides a method to reverse the impact of epigenetic silence in diseases where hypermethylation is a contributing factor [46]. Non-integrative delivery methods are clearly superior to viral vectors for CRISPR components, such as mRNA or ribonucleoprotein complexes. It is because they can minimize tumorigenicity risks [47].

Despite its promise, CRISPR-based epigenetic editing faces problems. For instance, Off-target effects occurring at non-target loci remain a concern, but the improved high-fidelity Cas9 variants (e.g., HiFi-Cas9) effectively mitigate it [48,49]. Delivery efficiency is transient, suggesting that the expression of CRISPR components in vivo is limited by cellular uptake barriers, thus it is necessary for the advanced delivery systems (e.g., lipid nanoparticles) [50,51]. Stability of epigenetic changes proves that the edited epigenetic states can revert over time, it is required for the repeated treatments or combinatorial approaches with small molecules (e.g., NAD^+^ boosters) [52].

### Ethical Considerations

The derivation of iPSCs typically involves the use of somatic cells, such as skin fibroblasts or blood cells, from humans. It is vital essential to guarantee that individuals donating their cells for reprogramming are fully informed about the procedure and any possible applications, and that their consents are given voluntarily and without coercion. Current guidelines, such as the ISSCR 2021 Standards, emphasize rigorous consent processes, ethical procurement, and donor autonomy. The focus is on oversight tiers for cell line procurement (Tiers 1-3) and compliance with original consent terms [53]. The NIH framework explicitly states consent for future genomic analyses and strict adherence to the approved secondary uses, even if the term "prohibits" is not directly used. Unapproved uses would violate consent agreements and regulatory standards [54].

Although iPSCs are a potent tool for research and therapy, the creation of human gametes for reproductive cloning is regarded as unethical in most cases [55]. The majority of the scientific and bioethical community supports the use of iPSCs for therapeutic purposes but strongly condemns reproductive cloning due to concerns about the safety and welfare of the offspring [56]. In order to handle these ethical issues, the establishment of institutional review boards and supervisory bodies guarantees the solution of iPSC research and applications [16,57].

Public engagement and education were also addressed in the ethical considerations of iPSCs. It was essential to initiate an open dialogue with the public regarding the potential benefits and risks of iPSC technology. It was also crucial to involve diverse viewpoints in the formulation of moral principles and policy decisions [26,58].

## Conclusion

iPSC and CRISPR technologies collectively present transformative strategies to delay aging and restore cellular vitality, paving the way for rejuvenation therapies. Future studies should focus on improving the reprogramming efficiency, minimizing the risk of tumorigenicity, and exploring the optimized CRISPR-based epigenetic editing technique.
